# Outcome of in hospital cardiac arrest (IHCA) in mechanically ventilated (MV) patients. A single center study

**DOI:** 10.1186/2197-425X-3-S1-A195

**Published:** 2015-10-01

**Authors:** M Ould Chikh, P Burtin, JY Bigeon, C Halchini, M Barral, A Roussiaux, P Courant

**Affiliations:** Clinique du Millénaire, Anesthésie Réanimation, Montpellier, France

## Introduction

Few studies are available on the outcome and prognosis factors of IHCA treated with cardiopulmonary resuscitation (CPR) in mechanically ventilated (MV) patients. MV is associated with increased mortality of IHCA in ICU patients [1] and in patients under anesthesia [2]. The only epidemiologic report show a decreased survival rate in MV patients without solving the bias related to illness severity or to indication for MV [3]. Authors of these reports did not comment on risk factors for mortality in MV patients.

## Objectives

The purpose of this study is to evaluate the outcome and risk factors for mortality in IHCA among MV patients.

## Methods

IHCA are defined by presence of chest compression and/or external electrical shock (EES). All IHCA witnessed by an anesthesiologist in the OR or ICU are analyzed. All IHCA are recorded on a database including : demographic data, medical history, location of IHCA, event time sequence of CPR, initial cardiac rhythm (VF, VT, PEA, Asystole), first treatment attempt, number of EES, type of IV treatment, immediate survival rate (ISR). For Survivors : SAPS II score, duration of ICU stay, treatments and hospital survival rate (HSR) are recorded. Two groups defined by presence (group V) or absence (Group NV) of MV prior to IHCA were compared.

## Results

From 01/01/2009 to 12/01/2014, 357 IHCA were recorded (5.2 IHCA/1000 stays) and 161 were analyzed (107 men ; mean age 69+/-12). Group V patients (n = 81; 50.3%) had significantly higher rate of epinephrine use (96.3% vs 57.5%), longer time to ROSC (7.81+/-6 vs 4.39+/-5.2 min), longer LOS in ICU (9.54+/-14 vs 5.28+/-6.33 days), lower ISR (59.26% vs 76.25%) and lower HSR (33.3% vs 63.75%) compared to NV patients (fig. 1) (p < 0.05). There was no differences between groups for age, sex ratio, comorbidity, circumstances, time to intervention (1.02+/-0.2 vs 1.08+/-0.3 min, NS), initial cardiac hythm.

## Conclusions

This is the first European study on this topic. This is also the first study controling patients for time to intervention and for MV indication (OR and ICU). Our results confirm a significant decrease in immediate and hospital survival rate among patients under MV at the time of the IHCA. These decrease cannot be relied to differences in illness severity or in initial care nor to initial cardiac rythm. Longer time to ROSC and higher rate of epinephrine use in MV patients strongly suggest that these patients may be resistant to vasopressors. These results also suggest that experimental datas [4] showing an adverse effect of MV on coronary and cerebral perfusion in a VF model may be clinically relevant. Cardiac arrest treated with CPR is one more situation in which cardiopulmonary interaction may affect outcome. A specific management of MV during IHCA treatment must be discussed.
